# Interpretable Machine Learning and Spatiotemporal Modeling of Meteorological and Environmental Drivers for Tuberculosis Incidence in China

**DOI:** 10.3390/toxics14060537

**Published:** 2026-06-21

**Authors:** Zihao Wang, Siyuan Li, Xiaotong Jiang, Kang Hu, Yangzhou Wu

**Affiliations:** 1 State Key Laboratory of Spatial Datum, College of Geographical Sciences, Henan University, Zhengzhou 450046, China; zihaowang@henu.edu.cn; 2 Shandong Key Laboratory of Eco-Environmental Science for the Yellow River Delta, Shandong University of Aeronautics, Binzhou 256603, China; 3 Jiangsu Collaborative Innovation Center of Atmospheric Environment and Equipment Technology, Nanjing University of Information Science & Technology, Nanjing 210044, China; 4 School of Environment and Architecture, University of Shanghai for Science and Technology, Shanghai 200093, China

**Keywords:** tuberculosis, meteorological drivers, air pollution, machine learning, Geographically and Temporally Weighted Regression (GTWR), spatial heterogeneity

## Abstract

Tuberculosis (TB) remains a major public health burden in China. Although meteorological and environmental factors are recognized to influence TB transmission, their non-linear effects and spatiotemporal heterogeneity have not been fully elucidated. Based on monthly TB incidence data from 31 provinces in China during 2005–2020, this study systematically investigated these effects by integrating nine meteorological and air pollution variables within a combined machine learning and spatial statistical modeling framework. The results indicated that the Extreme Gradient Boosting (XGBoost) model effectively captured the complex non-linear relationships between environmental exposure and TB incidence. SHAP interpretability analysis identified surface pressure (SP), vegetation coverage, and PM_2.5_ as the key drivers and revealed pronounced nonlinear response patterns and threshold effects. In particular, the promoting effect of PM_2.5_ on TB incidence increased sharply at medium-to-high concentration levels. To further investigate spatial and temporal non-stationarity, Geographically and Temporally Weighted Regression (GTWR) was applied. The results demonstrated strong spatiotemporal heterogeneity in driver effects across provinces. The influence of PM_2.5_ showed a consistently positive association with TB incidence and exhibited a distinct temporal evolution characterized by an initial strengthening before 2015 followed by a weakening thereafter, closely aligning with China’s air pollution control process. These findings provide new insights into the nonlinear and spatiotemporally heterogeneous effects of meteorological and environmental factors on TB incidence and support the development of more targeted, region-specific TB prevention strategies.

## 1. Introduction

Tuberculosis (TB), a chronic respiratory infectious disease caused by Mycobacterium tuberculosis, continues to pose a severe threat to global public health [1,2]. As a high-burden country, China has long prioritized understanding the epidemiological characteristics and driving mechanisms of TB within both academic and public health spheres [3]. In China, Bacille Calmette–Guérin (BCG) vaccination has long been integrated into the national routine immunization program for newborns, serving as a fundamental primary prevention measure to reduce the risk of severe childhood tuberculosis [4,5]. Previous research has predominantly focused on traditional determinants such as socioeconomic status [6,7], population mobility [8], and accessibility of healthcare services [9,10], establishing a theoretical framework to explain how these factors influence TB incidence risk [11]. However, as a respiratory infectious disease, the transmission process of TB and the viability of its pathogen are inevitably modulated by external meteorological and environmental conditions. In contrast to the extensive research on socioeconomic factors, the role of meteorological and environmental factors in TB epidemiology has not been systematically evaluated. Existing studies often treat meteorological indicators merely as auxiliary control variables, lacking in-depth exploration of multidimensional meteorological–environmental combinations and their complex interactions [12].

Evidence suggests that TB incidence exhibits distinct seasonal characteristics [13]. A national time-series study in China revealed a significant peak in TB notification rates during the spring of each year [14], a phenomenon also corroborated by regional studies in Henan Province [15]. Several studies have further indicated that incorporating meteorological factors into predictive models significantly enhances their predictive capacity [16], suggesting that meteorological conditions may alter the risk of human infection by influencing the survival and transmission dynamics of Mycobacterium tuberculosis in the environment. For instance, low temperatures tend to promote indoor crowding, thereby facilitating respiratory transmission, while lower wind speeds may impede the dispersion of tubercle bacilli [17].

In different regions, the association between meteorological factors and TB presents significant inconsistencies, highlighting the regional complexity and heterogeneity of these effects. For example, a study in Chennai, India, found associations between dew point temperature, ambient temperature, and the progression from latent to active TB, with these relationships varying across seasons [18]. In China, a study covering multiple cities in the southwest region reported complex non-linear relationships between temperature, relative humidity, and TB incidence risk, accompanied by lag effects [19]. Conversely, a national multi-center study revealed discrepancies at a more macro level, finding that higher monthly average wind speeds were associated with lower TB risk, while the relationship between precipitation and risk exhibited regional specificity [20]. These discrepancies collectively demonstrate that the impact of meteorological factors on TB is not universally uniform but is profoundly modulated by specific geographical, climatic, and socioeconomic contexts.

In terms of methodology, this field currently relies heavily on traditional statistical models or spatial statistical tools such as Moran’s I [21]. While these methods possess advantages in identifying linear relationships or spatial differentiation, they struggle to capture the complex non-linear relationships that likely exist between meteorological–environmental factors and TB incidence. In recent years, a few studies have attempted to introduce machine learning algorithms. However, most applications remain shallow, often employing single models focused primarily on predictive performance and relying on traditional feature importance rankings for interpretation, failing to deeply reveal the internal decision-making mechanisms of the models [22,23]. In particular, powerful ensemble learning algorithms like XGBoost remain underutilized in domestic research, and their potential value has not been fully exploited.

Regarding spatial scale, existing studies are often confined to single provinces or specific regions, lacking comprehensive national-level analysis, which makes it difficult to reveal macro-level meteorological laws. Conversely, national aggregate analyses, while capturing overall trends, generally overlook the spatial heterogeneity of meteorological impacts at the provincial scale, potentially leading to the “ecological fallacy” [24,25].

To simultaneously address the dual challenges of macro-pattern identification and local characterization, we developed an integrated framework coupling Extreme Gradient Boosting (XGBoost) and Geographically and Temporally Weighted Regression (GTWR). On one hand, leveraging the robust non-linear fitting capability of XGBoost, this study robustly extracts key drivers and their common influence patterns from national data. On the other hand, building upon this, the GTWR model is introduced to precisely quantify the dynamic evolution of the regression coefficients of these key factors across geographical space and time, thereby fundamentally revealing the actual geographical patterns and spatiotemporal differentiation mechanisms of their effects. Specifically, this study aims to: (1) identify key meteorological–environmental factors and determine their relative importance at the national macro level; (2) deeply reveal the specific interaction modes and non-linear relationships between key factors and incidence rates; and (3) focus on analyzing the spatial heterogeneity of these meteorological effects across different provinces. This research constructs a progressive chain of evidence moving from macro-pattern identification to mechanistic explanation and finally to spatial heterogeneity analysis, providing comprehensive and refined insights into the role of meteorological–environmental conditions in TB transmission.

## 2. Materials and Methods

### 2.1. Data Sources

This study utilized monthly panel data from 31 provincial-level administrative divisions in mainland China, covering the period from January 2005 to December 2020. The dataset comprises three categories: tuberculosis (TB) incidence, meteorological factors, and environmental factors.

Specifically, TB incidence data were obtained from the Public Health Science Data Center [26]. Meteorological factors—including temperature, U-component of wind (east–west), V-component of wind (north–south), surface pressure (SP), high vegetation cover (HVC), average surface downward UV radiation, and relative humidity (RH)—were derived from the ERA5 reanalysis dataset produced by the European Centre for Medium-Range Weather Forecasts (ECMWF) [27]. Environmental factors, ambient PM_2.5_ concentration data were sourced from the SatPM_2.5_ dataset developed by Washington University [28]. Ozone (O_3_) concentration data were obtained from the ECMWF Atmospheric Composition Reanalysis 4 (EAC4) dataset [29].

These multi-source datasets differ in original spatial and temporal resolutions. To construct a unified provincial-monthly analytical dataset consistent with TB incidence records, we performed a standardized spatiotemporal harmonization procedure for all variables. Specifically, the ERA5 reanalysis dataset has a spatial resolution of 0.25° × 0.25° and an hourly temporal resolution; the SatPM_2.5_ dataset provides annual-average concentrations at a spatial resolution of 0.01° × 0.01°; and the EAC4 atmospheric composition reanalysis dataset, which serves as the source of ozone data, has a spatial resolution of 0.75° × 0.75° with a 3 h temporal interval. Spatially, all gridded meteorological and environmental variables were aggregated to the provincial administrative scale using area-weighted averaging to match the statistical unit of health data. Temporally, all variables were processed into monthly mean values to ensure uniform temporal resolution across the entire dataset.

After the spatiotemporal harmonization process, a complete provincial-monthly panel dataset was constructed as the training dataset for subsequent machine learning models. Table 1 presents the descriptive statistics of all variables in the model training dataset, covering minimum, maximum, mean and standard deviation values, to demonstrate the basic distribution characteristics of the input variables.

The nine meteorological and environmental variables were selected based on a synthesis of the most commonly reported and representative influencing factors in previous studies.

### 2.2. Analytical Framework

**Figure 1:**
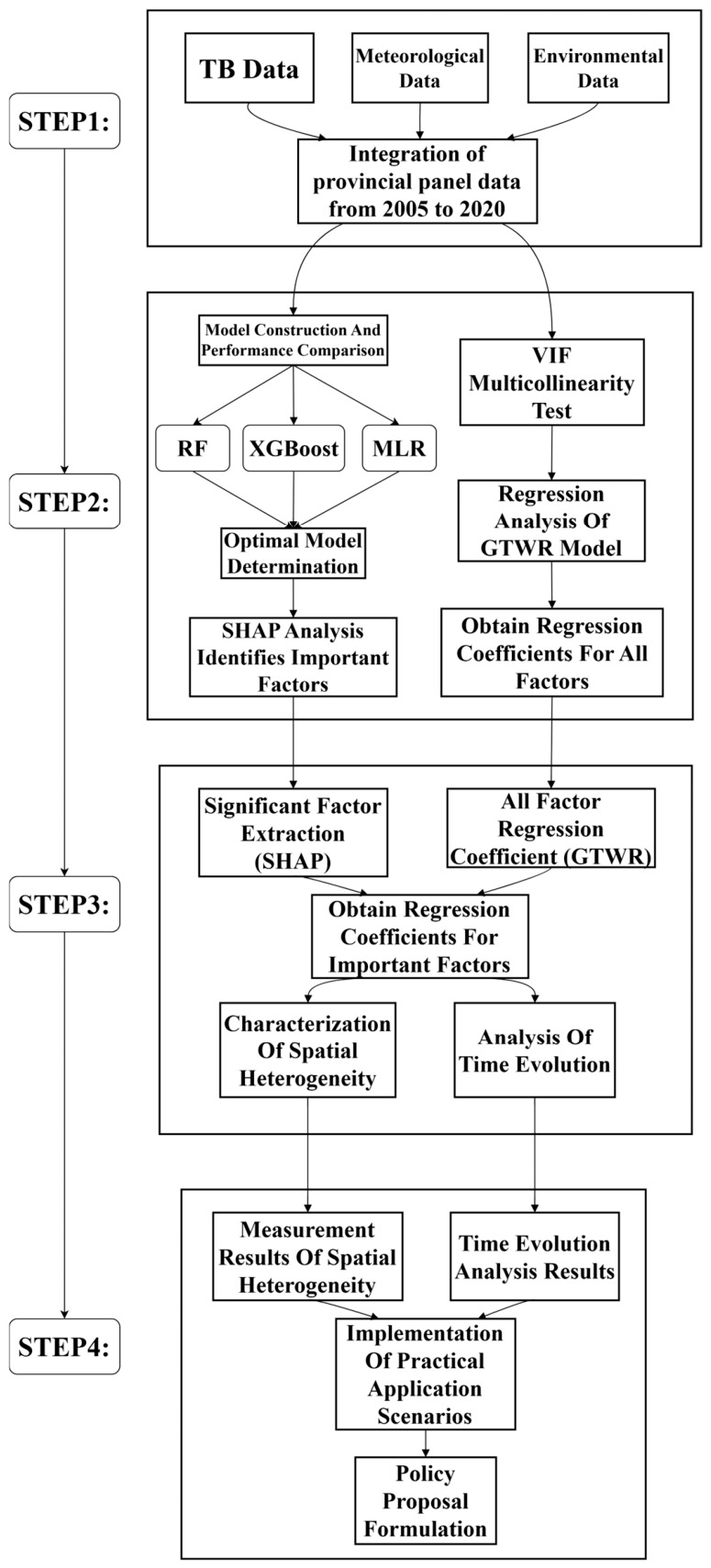
Flowchart of the progressive analysis framework for meteorological–environmental driving effect research.

This study employed a progressive analytical framework comprising three components: “Model Selection, Mechanism Elucidation, and Spatiotemporal Heterogeneity Detection.” First, three predictive models—Multivariate Linear Regression (MLR), Random Forest (RF), and XGBoost—were constructed, and the optimal model was selected based on fitting performance comparison. Second, utilizing the optimal model, the SHAP (SHapley Additive exPlanations) interpretability framework was introduced to evaluate the global contribution of each meteorological–environmental factor. This step aimed to screen for key drivers and deeply reveal the complex non-linear relationships between these drivers and TB incidence. Finally, the GTWR model was applied to finely characterize the effects of key factors, analyzing their spatial heterogeneity at the provincial scale and their dynamic evolution over time. This integrated system achieved a comprehensive chain of analysis, progressing from global pattern discovery to local mechanism resolution and finally to spatiotemporal disparity profiling. The overall research workflow, encompassing data collection, preprocessing, model construction, and result interpretation, is illustrated in Figure 1.

### 2.3. Construction and Comparison of Predictive Models

#### 2.3.1. Multivariate Linear Regression Model

Multivariate Linear Regression (MLR) was selected as the baseline model, assuming a linear relationship between meteorological factors and TB incidence. The model is expressed as:

(1) $$y_{i}=\beta_{0}+\sum_{k=1}^{p}\beta_{k}x_{ik}+\varepsilon_{i}$$

where $y_{i}$ is the TB incidence of the i-th sample, $\beta_{0}$ is the intercept, $\beta_{k}$ is the regression coefficient for the k-th meteorological factor, $x_{ik}$ is the standardized value of the k-th factor, and $\varepsilon_{i}$ is the random error term (following a normal distribution).

#### 2.3.2. Random Forest Model

Random Forest (RF) is based on ensemble learning principles. It generates B training subsets using bootstrap aggregating (Bagging). A decision tree is constructed for each subset, with a random subset of features selected for node splitting. The final prediction is the mean of all decision trees:

(2) $$\hat{y}=\frac{1}{B}\sum_{b=1}^{B}\;T_{b}\left(x\right)$$

where $\hat{y}$ is the predicted value, B is the number of decision trees, and $T_{b}(x)$ is the prediction result of the b-th tree. This dual randomness design effectively reduces the risk of overfitting.

#### 2.3.3. XGBoost Model

The XGBoost (Extreme Gradient Boosting) model is an implementation of the gradient boosting decision tree algorithm. It iteratively adds weak learners (decision trees) to minimize an objective function, which consists of a loss function and a regularization term to balance model fit and complexity:

(3) $$Obj(\theta )=\sum_{i=1}^{n}\;L(y_{i},{\hat{y}}_{i})+\sum_{k=1}^{K}\;\Omega (f_{k})$$

where $L(y_{i},{\hat{y}}_{i})$ is the squared loss function (measuring the difference between predicted and actual values), and $\Omega (f_{k})=\gamma T+\frac{1}{2}\lambda ∥w{∥}^{2}$ is the regularization term (where T is the number of leaves, w is the vector of leaf weights, and γ and λ are penalty coefficients). An additive training strategy is employed to fit the residuals.

The dataset was randomly split into a training set (80%) and a test set (20%) to evaluate model performance. Detailed descriptions of model hyperparameter tuning procedures and additional performance results are provided in Section S1 (Tables S1 and S2).

We note that tuberculosis has a long incubation period and seasonal transmission patterns, which may warrant the use of lagged meteorological and environmental variables. In this study, we considered including lag features (e.g., 1-, 2-, and 3-month lags) during preliminary analyses. However, we ultimately chose not to include them because our primary objective was to identify contemporaneous nonlinear relationships and spatiotemporal heterogeneity rather than to optimize pure time-series prediction. Moreover, adding multiple lag terms for autocorrelated variables would increase model complexity and the risk of multicollinearity, potentially compromising the interpretability of SHAP dependence plots.

#### 2.3.4. Model Interpretability Analysis (SHAP Framework)

To demystify the “black-box” nature of machine learning models, this study introduced the SHAP framework based on cooperative game theory to quantify the contribution logic of each feature to the prediction result. The core principle of SHAP is derived from Shapley values, which essentially distribute the “total contribution of the feature set to the model prediction” fairly among individual features. The core formula is:

(4) $$\phi_{i}=\sum_{S\subseteq N\setminus i}\;\frac{\left|S\right|!\left(\left|N\right|-\left|S\right|-1\right)!}{\left|N\right|!}\left[f_{x}\left(S\cup i\right)-f_{x}\left(S\right)\right]$$

where $\phi_{i}$ is the SHAP value of the i-th meteorological factor (positive/negative values correspond to promoting/inhibiting the predicted incidence, and the absolute value reflects the intensity of the effect); N represents the set of all factors; S represents any subset excluding the i-th factor; $f_{x}(S)$ represents the prediction result using only the factors in subset S; and $f_{x}(S\cup i)$ is the prediction output after adding the i-th feature. The weighting coefficient $\frac{|S|!(|N|-|S|-1)!}{|N|!}$ ensures unbiased contribution allocation.

All data preprocessing, MLR, Random Forest, XGBoost, and SHAP analyses were implemented using Python (version 3.9). The main Python packages used were: pandas and numpy for data manipulation; scikit-learn for MLR and Random Forest; xgboost for the XGBoost model; and shap for SHAP interpretability.

### 2.4. Spatiotemporal Heterogeneity Analysis

To deeply reveal the spatiotemporal differentiation patterns of key influencing factors on TB incidence, this study adopted the Geographically and Temporally Weighted Regression (GTWR) model as the core analytical tool to systematically explore the spatial differences and temporal evolution of factor intensities. Considering that GTWR is sensitive to multicollinearity among independent variables, a Variance Inflation Factor (VIF) analysis was first performed to verify data reliability. Subsequently, the model was fitted based on standardized data to quantify the coefficients for each province and year, providing a quantitative basis for subsequent spatial heterogeneity and mechanistic analysis.

#### 2.4.1. Multicollinearity Test of Influencing Factors

To prevent coefficient distortion in the GTWR model caused by multicollinearity, a VIF test was conducted for the influencing factors:

(5) $$VIF_{j}=\frac{1}{1-R_{j}^{2}}$$

where $VIF_{j}$ is the variance inflation factor for the j-th factor, and $R_{j}^{2}$ is the coefficient of determination obtained by regressing that factor against all other factors. The widely accepted academic threshold of *VIF* < 5 was adopted to judge the absence of significant multicollinearity [30].

#### 2.4.2. GTWR Model

To capture the spatial non-stationarity and temporal dynamics of meteorological and environmental impacts, the GTWR model was employed. Its basic form is:

(6) $$y_{i}=\beta_{0}(u_{i},v_{i},t_{i})+\sum_{k=1}^{p}\;\beta_{k}(u_{i},v_{i},t_{i})x_{ik}+\varepsilon_{i}$$

where $\left(u_{i},v_{i}\right)$ denotes the longitude and latitude of the provincial administrative center for the i-th sample, $t_{i}$ is the time variable (month), and $\beta_{k}(u_{i},v_{i},t_{i})$ represents the dynamic regression coefficient varying with spatial location and time, reflecting the intensity and direction of meteorological and environmental influences across different provinces and periods. The model uses a Gaussian kernel function to calculate spatiotemporal distance weights between samples, adaptively adjusting the range of influence. The weight function is:

(7) $$W_{ij}=exp\left(-\frac{d_{ij}^{2}}{h^{2}}\right)$$

where $d_{ij}$ is the spatiotemporal distance between sample i and j (a weighted integration of spatial and temporal distances), and h is the bandwidth parameter (optimized via cross-validation). To visually present the results, four representative cross-sectional time points—2005, 2010, 2015, and 2020—were selected. The regression coefficients output by the GTWR model were visualized using thematic maps to demonstrate the spatial differentiation and spatiotemporal evolution patterns of the meteorological drivers.

GTWR modeling was performed using ArcGIS 10.8, specifically its built-in GTWR tool, which is designed to calibrate spatially and temporally varying regression coefficients.

## 3. Results

### 3.1. Comparison of Predictive Model Performance

To systematically evaluate the performance of different models in characterizing the relationship between meteorological factors and TB incidence, this study conducted a comprehensive comparison of the predictive capabilities of Multivariate Linear Regression (MLR), Random Forest (RF), and XGBoost. The results indicated that machine learning algorithms significantly outperformed the traditional linear model, capturing the complex inter-variable relationships more effectively.

**Figure 2:**
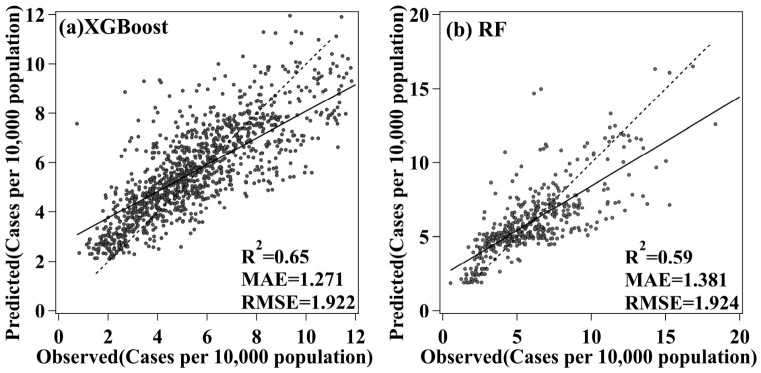
Scatter plot comparing observed and predicted values on the test set for the XGBoost (**a**) and Random Forest (**b**) models. The solid line denotes the fitted regression curve; the dashed line is the 1:1 reference line.

Specifically, the coefficient of determination ($R^{2}$) for the MLR model was only 0.20 (Table S3), with detailed residual statistics provided in Table S4. Although this result is consistent with the common explanatory power of linear models in previous studies (typically $R^{2}$ ranges between 0.20 and 0.35), it clearly reveals inherent limitations in adequately representing potential interactions and non-linear associations among variables. In contrast, both ensemble learning-based machine learning models improved prediction accuracy by approximately three times (Figure 2), confirming the non-linear nature of the research problem.

Further comparison between the two machine learning models revealed that the XGBoost model achieved the superior performance on the test set, with an $R^{2}$ of 0.65, a Mean Absolute Error (MAE) of 1.271, and a Root Mean Square Error (RMSE) of 1.922. The Random Forest model performed slightly less well, with an $R^{2}$ of 0.59, and MAE and RMSE values of 1.381 and 1.924, respectively. XGBoost not only demonstrated superior goodness-of-fit but also yielded lower overall prediction errors, indicating more robust predictive capability. Its superior comprehensive performance provides a more reliable methodological basis for the subsequent in-depth assessment of factor importance and mechanism analysis. Consequently, the following analyses in this study are primarily based on the XGBoost model.

It should be noted that the random training–test split used in this study may introduce a certain degree of spatiotemporal information leakage. Future studies should adopt more rigorous validation strategies, such as temporal and spatial cross-validation, together with larger spatiotemporal datasets, to provide a more robust assessment of model generalizability and potentially further improve predictive performance.

### 3.2. Evaluation of Importance and Mechanism Analysis of Meteorological–Environmental Factors Based on SHAP

To systematically assess the relative importance of various meteorological and environmental factors on TB incidence and to deeply analyze their influence directions and mechanisms, this study employed the SHAP interpretability framework for global feature contribution analysis. By calculating the mean absolute SHAP values for each factor, a stable importance ranking was obtained, providing a reliable basis for identifying key drivers.

**Figure 3:**
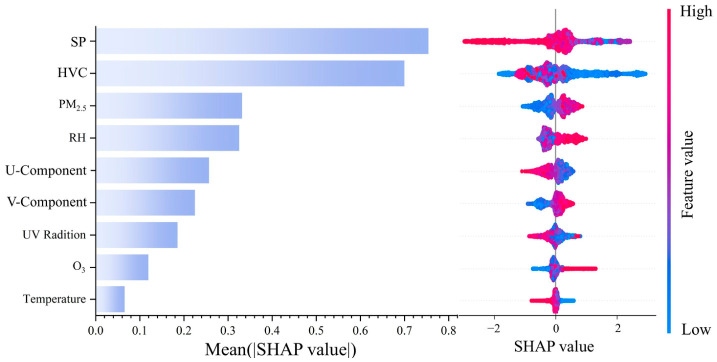
Global importance and influence direction of meteorological–environmental factors based on SHAP values. Beeswarm plot illustrating the distribution of SHAP values for each factor and its corresponding influence direction on tuberculosis incidence; Bar plot showing the overall importance ranking of factors based on mean absolute SHAP values.

As shown in Figure 3, the variables are ranked by their global importance as follows: Surface Pressure (SP) > High Vegetation Cover (HVC) > PM_2.5_ > Relative Humidity (RH) > U-component of wind > V-component of wind > Surface Downward UV Radiation > O_3_ > Temperature. SP emerged as the most prominent predictor, with a mean SHAP value significantly exceeding other factors, suggesting that atmospheric pressure conditions may be a key meteorological element regulating TB incidence risk. HVC and PM_2.5_ ranked second and third, respectively, with similar importance levels that were notably higher than subsequent factors, highlighting the critical roles of ecological vegetation conditions and particulate pollution in disease mechanisms.

Regarding the direction of influence, the SHAP values for SP were primarily distributed in the negative range. Higher pressure values (red dots in the figure) corresponded to more negative SHAP values, indicating that higher surface pressure has a sustained inhibitory effect on TB incidence. This finding resonates with a study on TB pathogenesis in Northwest China, which also confirmed atmospheric pressure as a significant meteorological factor influencing TB transmission [31]. The potential mechanisms by which surface pressure affects TB are complex. On one hand, higher pressure conditions are typically associated with stable meteorological conditions, which may limit the dispersion range of droplet nuclei carrying Mycobacterium tuberculosis in the air. On the other hand, early scientific discourse on the relationship between the atmospheric environment and TB systematically explored the impact of different pressure environments [32], noting that pressure changes might participate in the disease process by altering the physiological state of the human respiratory system and the microenvironment for pathogen survival.

The SHAP values for HVC were also predominantly negative, indicating that increased vegetation coverage exerts an inhibitory effect on TB incidence. This finding aligns with conclusions from recent studies in environmental health, suggesting that vegetation can prevent respiratory infectious diseases through multiple pathways. Specifically, high vegetation coverage can significantly improve regional air quality; trees and plants act as natural filters, effectively adsorbing and removing pollutants such as PM_2.5_ from the air [33]. Furthermore, volatile organic compounds (VOCs) released during photosynthesis help increase the concentration of negative oxygen ions in the atmosphere [34,35,36]. These ions have been confirmed to improve alveolar ventilation function, enhance the clearance efficiency of respiratory mucosal cilia, and create an unfavorable environment for the survival of Mycobacterium tuberculosis. In densely populated urban areas, high vegetation coverage not only reduces pathogen transmission probability by lowering local pollutant concentrations but also provides green spaces for outdoor physical activities and social interactions. This helps alleviate mental stress and enhance physical immunity, thereby indirectly reducing TB incidence risk [37]. Notably, a multi-center ecological study in China further indicated that the inhibitory effect of vegetation on TB is more significant in areas with complete green space structure and high connectivity [33], suggesting that future urban planning should prioritize spatial configuration and structural optimization in addition to vegetation quantity.

Conversely, the SHAP values for PM_2.5_ were concentrated in the positive range. As concentrations increased (red dots), the positive contribution continuously strengthened, clearly indicating that PM_2.5_ exposure is a significant risk factor for TB incidence. This is consistent with findings that both short-term and long-term pollution exposure increase the risk of TB occurrence and mortality [38], implying that the health hazards of PM_2.5_ may exhibit an accelerated cumulative effect under high-pollution scenarios. Biologically, evidence suggests that PM_2.5_ can weaken the body’s resistance to M. tuberculosis by inducing chronic airway inflammation, reducing macrophage phagocytic capacity, and disrupting alveolar immune homeostasis [39,40]. Simultaneously, particulate matter acting as a potential “aerosol carrier” may prolong the survival time of pathogens in the air and facilitate spatial transmission, further elevating infection risk [41,42,43]. Additionally, the effect of PM_2.5_ may interact with meteorological conditions; for instance, pollution accumulation caused by winter inversions combined with poor ventilation and increased indoor confinement can jointly amplify infection risk.

Among the remaining factors, RH and the U-component of wind showed moderate importance, but their SHAP value distributions were relatively concentrated, indicating that their influence intensity and stability were lower than the three key factors mentioned above. The mean SHAP values for the V-component of wind, UV radiation, ozone concentration, and temperature were relatively low, suggesting their overall contribution to incidence rates on a global scale is limited, although they may still exert regulatory effects in specific regions or value ranges.

In summary, based on the SHAP interpretability method, this study identified Surface Pressure, High Vegetation Cover, and PM_2.5_ as the three key drivers affecting TB incidence in China and clarified their directions of action and basic mechanisms. These results not only resolve the “black-box” issue of machine learning models but also lay a solid foundation for the subsequent in-depth analysis of the non-linear effects and spatiotemporal heterogeneity of these key factors.

### 3.3. Analysis of Non-Linear Effects and Threshold Effects

To elucidate the specific influence patterns of key drivers, we constructed SHAP dependence plots to characterize nonlinear associations and identify critical thresholds that define the shifting impact of each factor on TB risk.

**Figure 4:**
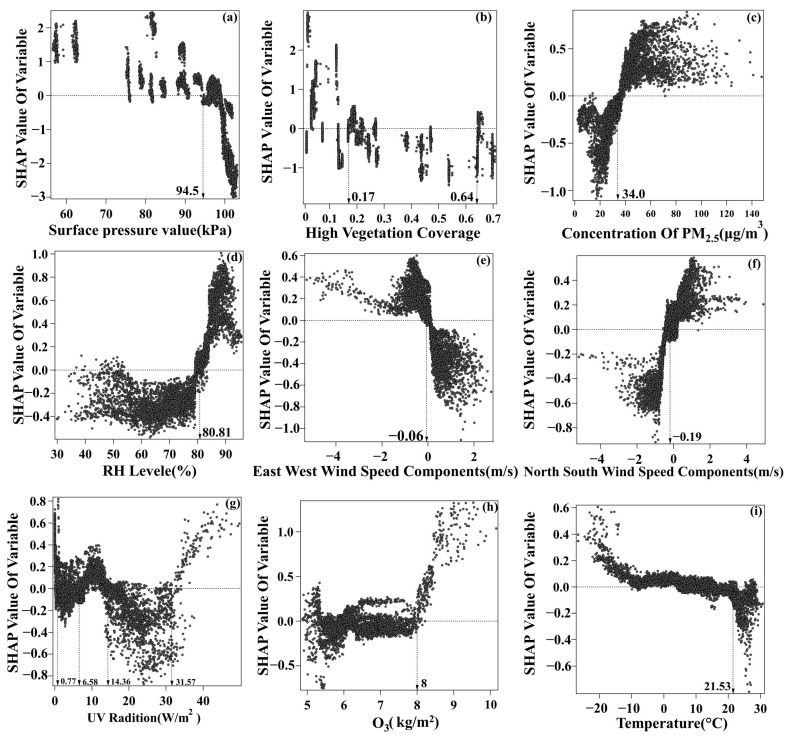
SHAP dependence plot of meteorological–environmental factors and TB incidence. (**a**) Surface pressure (SP); (**b**) High vegetation cover (HVC); (**c**) PM_2.5_; (**d**) Relative humidity (RH); (**e**) U-component of wind; (**f**) V-component of wind; (**g**) UV radiation; (**h**) O_3_; (**i**) Temperature.

As shown in Figure 4a, the relationship between SP and TB risk exhibits a continuous downward trend with a distinct inflection point at 94.5 kPa. Below this threshold, SHAP values are positive, though they decrease gradually as pressure rises. Once SP exceeds the 94.5 kPa, the SHAP values decline sharply into the negative range, indicating that the inhibitory effect of atmospheric pressure on TB transmission amplifies significantly. Higher surface pressure typically implies a more stable atmospheric stratification and weaker air convection. Such meteorological conditions may be unfavorable for the prolonged suspension and long-distance dispersion of droplet nuclei carrying M. tuberculosis, thereby reducing population exposure and infection risk [44,45]. This finding corresponds with previous study on TB pathogenesis in Northwest China, which identified atmospheric pressure as an important meteorological factor and noted the complex interaction between meteorological conditions and air pollution [31]. We further reveal the specific concentration threshold and non-linear characteristics of the inhibitory effect generated by SP.

As shown in Figure 4b, a distinct negative non-linear relationship exists between HVC and TB incidence risk. When vegetation cover is below 0.17, SHAP values are predominantly positive. Once vegetation cover exceeds the critical threshold of 0.17, SHAP values turn negative and decline further with increased coverage, indicating a stable and enhanced protective effect. This suggests a significant “activation threshold” for the vegetation; it suggests that the health benefits of green spaces only manifest continuously after crossing this critical point.

The impact of PM_2.5_ concentration on TB risk demonstrates a distinct threshold-triggered escalation, as depicted in Figure 4c. At concentrations below 34 μg/m^3^, SHAP values are generally negative, indicating that at a lower pollution level, the contribution of PM_2.5_ to TB incidence is relatively weak. However, once the concentration exceeds the turning point of 34 μg/m^3^, SHAP values shift rapidly to the positive range and show an approximately linear increasing trend with rising concentration. This indicates that the promoting effect of PM_2.5_ on TB incidence amplifies sharply at medium-to-high air pollution levels. Note that the apparent threshold of approximately 34 μg/m^3^ is a model-derived pattern from SHAP dependence analysis of the current dataset. It reflects nonlinear statistical associations learned by the model rather than a causal or mechanistic relationship, and should not be interpreted as a definitive biological threshold without independent epidemiological validation.

It is worth noting that the health risk threshold of 34 μg/m^3^ identified in this study is lower than China’s current annual mean PM_2.5_ standard (35 μg/m^3^; daily mean 75 μg/m^3^), yet significantly higher than the stricter annual guideline value of 25 μg/m^3^ proposed by the World Health Organization (WHO). Consistent with previous studies, [46,47], our findings demonstrate that residual TB risks persist even as national pollution levels decline. This suggests that simply meeting current national air quality standards may be insufficient to fully mitigate the environmental triggers of TB. Consequently, this study provides a critical scientific impetus for more ambitious emission reduction targets: transitioning beyond national compliance toward the stricter WHO health guidelines is essential to effectively safeguard public health against air pollution-sensitive infectious diseases. Corresponding SHAP dependence plots for the remaining factors are presented in Figure 4d–i.

### 3.4. Spatiotemporal Heterogeneity Results

#### 3.4.1. VIF Investigation Results

A Variance Inflation Factor (VIF) test was conducted on the nine influencing factors involved in this study, with results presented in Table 2. The test revealed that the VIF value for temperature was 6.023, exceeding the commonly accepted threshold of 5.0. Temperature is an important factor associated with TB epidemiology; however, monthly average temperature was excluded from the GTWR analysis due to multicollinearity (VIF > 5) to ensure model stability and reliable coefficient estimation. Its effects may be partially captured by correlated variables such as humidity and surface pressure. In addition, the use of monthly averages may not fully capture short-term temperature variability that could be relevant to transmission dynamics. After excluding this factor, the VIF values for the remaining eight influencing factors ranged between 1.597 and 2.642, all below the threshold of 5.0. This result indicates that there is no significant multicollinearity among the remaining variables, and they can be considered mutually independent. Consequently, the estimated effects of each factor on TB incidence are unlikely to be seriously distorted. This verification ensures that the independent contribution of each influencing factor to TB incidence is accurately quantified.

#### 3.4.2. Spatiotemporal Heterogeneity Analysis via GTWR

To accurately evaluate the spatiotemporal variation characteristics of meteorological impacts on TB incidence, a Geographically and Temporally Weighted Regression (GTWR) model was constructed using Z-score standardized data. The model fitting results showed a coefficient of determination ($R^{2}$) of 0.76 and a Corrected Akaike Information Criterion (AICc) value of 8720.16 (Table S5). The standardized residual distribution of the GTWR model is displayed in Figure S1. These metrics indicate that the GTWR model explains 76% of the spatiotemporal variance in TB incidence in China, demonstrating excellent fitting performance and explanatory power. This provides a reliable modeling foundation for the subsequent analysis of the spatial differentiation and temporal evolution of factor effects.

Based on the statistical results of the GTWR model regression coefficients for the entire period (Table 3), all meteorological and environmental factors exhibited distinct spatial variation characteristics. The coefficients for each factor spanned a wide range. For instance, the coefficient for SP fluctuated between −2.61 and 0.39; HVC ranged from −1.13 to 3.25. Such substantial variations in coefficient polarity and magnitude indicate that a single factor can act as a positive driver in certain regions while functioning as an inhibitor in others. These discrepancies highlight the spatial diversity of the mechanisms driving TB incidence. The varying intensity and direction of these effects across provinces reveal the presence of regionally dominant factors, suggesting that localized environmental conditions significantly modulate TB transmission dynamics.

The mean coefficient for SP was −0.70, indicating an overall inhibitory effect. The mean coefficient for HVC was −0.20, suggesting an overall protective effect, while PM_2.5_ yielded a mean coefficient of 0.19, indicating a consistent positive association with TB incidence across the study area. To further clarify the specific spatial patterns and temporal evolution trends of these key factors, the following sections provide a detailed analysis combined with visualization results, offering more refined scientific evidence for identifying regional priorities in public health prevention and control.

#### 3.4.3. Spatiotemporal Evolution Patterns of Key Factor Effects

**Figure 5:**
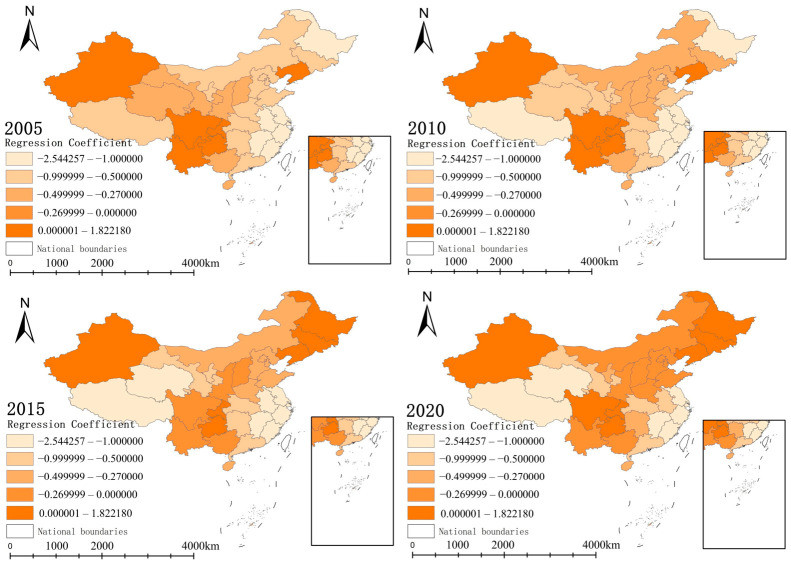
Spatiotemporal evolution of GTWR coefficients for surface pressure (SP) during 2005––2020.

To further examine the dynamic effects of SP (the primary meteorological driver) and PM_2.5_ (the primary environmental driver), four representative years (2005, 2010, 2015, and 2020) were selected for cross-sectional analysis. Combining these with the spatial distribution of coefficients output by the GTWR model, we analyzed their influence mechanisms across both spatial differentiation and temporal evolution (Figure 5).

In the temporal dimension, the influence of SP between 2005 and 2020 presented a dual characteristic of intensity adjustment and polarity shift. From 2005 to 2010, the inhibitory effect in North China and Inner Mongolia weakened. A critical transition occurred in 2015, as coefficients in Northeast China shifted from negative to positive, indicating the emergence of a promoting effect. By 2020, the concentration of high values in the northern regions became more prominent, and the promoting effect in Northeast China strengthened significantly.

Spatially, the distribution of SP regression coefficients exhibited distinct evolutionary features. In 2005, high-value zones were primarily concentrated in the Southwest and parts of the Northwest, forming a significant regional high-value belt. By 2010, this high-value zone contracted, and the inhibitory effect in North China further diminished. By 2015 and 2020, the high-value areas migrated toward Northern China, and the Northeast completed its transition from inhibition to promotion. These patterns suggest that the influence of SP on TB incidence undergoes a geographic migration from west to east, accompanied by an increasing positive regulatory role in northern latitudes.

**Figure 6:**
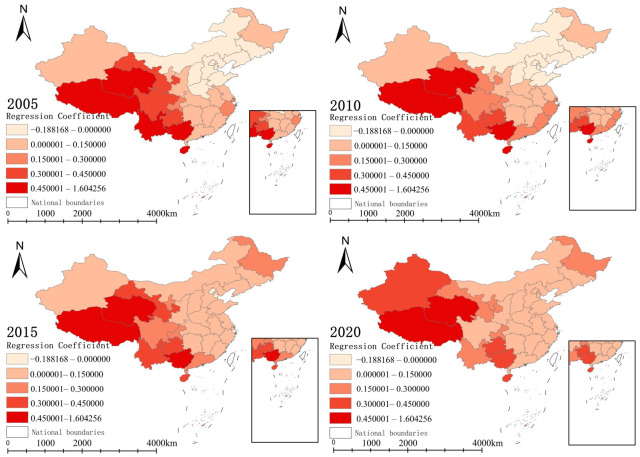
Spatiotemporal evolution of GTWR coefficients for PM_2.5_ during 2005–2020.

Figure 6 reveals strong spatiotemporal non-stationarity in the relationship between PM_2.5_ concentration and TB incidence. This implies that the increase in TB incidence caused by each unit increase in PM_2.5_ differs distinctively across regions and years. Spatially, we observed a clear “West-High, East-Low” pattern in the promoting effect of PM_2.5_. Specifically, regression coefficients in parts of the Southwest and Northwest were significantly higher than those in eastern coastal provinces. This indicates that TB incidence in these high-coefficient regions is more sensitive to changes in PM_2.5_. This spatial disparity may be related to imbalances in socioeconomic status and medical resources. The eastern coastal regions possess developed economies, robust medical security systems, and generally better population nutritional status and immunity, which may buffer the health shocks caused by environmental pollution. In contrast, the relatively scarce medical resources and weaker population resilience to environmental risks in the underdeveloped western regions amplify the pathogenic risk of PM_2.5_.

Temporally, from 2005 to 2020, the GTWR coefficients for PM_2.5_ evolved from initial regional differentiation to positive values across the entire domain, with influence intensity showing dynamic evolution. For instance, coefficients in the Xinjiang region gradually shifted from the median range in 2005 to the high-value range, reflecting a continuously strengthening driving effect of PM_2.5_ on TB incidence over time. Coefficients in the Northeast, North China, and Central regions rose from the low-value range in 2005 to the medium-high range; this change is likely closely related to the cumulative nature of pollution exposure in these traditional industrial hubs and the superimposed TB transmission risks caused by population agglomeration [48,49]. Conversely, coefficients in the southeast coastal region continuously declined from the medium-high range in 2010 to the medium-low range. This weakening trend is directly associated with the buffering effects formed by the early implementation of joint air pollution prevention and control policies since 2013 and the sustained input of regional public health resources [50,51]. Overall, this spatiotemporal evolution process indicates that although China has achieved significant improvements in air quality following the implementation of the “Clean Air Action Plan,” the positive driving effect of PM_2.5_ on TB incidence remains persistent. This also reflects the regional imbalance between environmental governance and public health interventions, warranting widespread attention.

## 4. Conclusions

This study developed an integrated framework—combining machine learning, interpretability analysis, and spatial econometrics—to elucidate the complex nonlinear and spatiotemporal drivers of tuberculosis in China. Our findings demonstrate that advanced machine learning can effectively capture the complex nonlinear relationships between TB incidence and meteorological–environmental factors while further accurately quantifying critical thresholds such as the 34 μg/m^3^ PM_2.5_ tipping point that triggers excess TB risk. Furthermore, the GTWR model revealed significant spatiotemporal non-stationarity among primary drivers The reversing polarity of SP effects in the Northeast and the shifting intensity of PM_2.5_ impacts highlight the urgent need to replace static national protocols with dynamic, regionalized strategies informed by localized environmental risks.

Based on these findings, we recommend the implementation of regionally differentiated prevention and control strategies. In high-pollution regions, it is imperative to recognize air quality improvement as a critical component of TB control, fostering policy synergy between environmental governance and public health sectors. Simultaneously, dynamic risk early warning platforms should be established at the provincial level, incorporating critical PM_2.5_ environmental thresholds into real-time warning indicators to promote cross-departmental data integration.

Overall, this study overcomes the limitations of previous research by capturing the spatiotemporal coupling of meteorological and environmental factors. This study is based on province-level aggregated data. Therefore, the findings reflect population-level associations rather than individual-level causal relationships. Although recent studies have also reported significant statistical associations between atmospheric pressure and TB incidence [31,52], the underlying physiological mechanisms remain speculative. Similarly, vegetation coverage has also been linked to improved air quality and respiratory function [53,54], but the pathways linking these factors to TB risk are still unclear. These interpretations should therefore be considered hypothesis-generating and require further investigation.

Future research should further integrate broader multi-dimensional determinants, such as socioeconomic factors and population mobility, to construct finer-scale explanatory frameworks at the city or county level, thereby continuously enhancing the comprehensive understanding of TB pathogenesis.

## Figures and Tables

**Table 1:** Descriptive statistics of variables in the model training dataset.

Variable	Unit	Minimum	Maximum	Mean	Standard Deviation
UV Radiation	W/m^2^	0.01	49.77	11.86	10.02
U-component of wind	m/s	−5.28	2.75	−0.07	0.86
V-component of wind	m/s	−5.20	4.92	−0.10	0.87
Temperature	°C	−26.70	30.7	9.93	12.38
SP	kPa	56.53	103.14	91.79	10.95
HVC	/	0.01	0.71	0.23	0.20
PM_2.5_	μg/m^3^	2.16	147.60	39.58	20.28
O_3_	kg/m^2^	4.87	10.16	6.42	0.79
RH	%	29.85	95.58	73.55	13.26

**Table 2:** VIF test results of meteorological–environmental factors.

Variable	VIF Value	Variable	VIF Value
HVC	1.597	UV Radiation	2.245
V-component of wind	1.696	O_3_	2.301
PM_2.5_	2.110	U-component of wind	2.352
RH	2.299	SP	2.642

**Table 3:** Descriptive statistics of regression coefficients of each factor in the full-period GTWR model.

	Mean	Min	Lower Quartile	Median	Upper Quartile	Max
Intercept	0.61	−1.12	0.08	0.41	1.02	6.01
SP	−0.70	−2.61	−1.78	−0.63	−0.29	0.39
HVC	−0.20	−1.13	−0.91	−0.05	0.36	3.25
PM_2.5_	0.19	0.01	0.06	0.18	0.33	0.63
RH	0.26	0.03	0.15	0.19	0.37	0.72
U-component of wind	−0.16	−0.87	−0.19	−0.14	0.04	0.26
V-component of wind	−0.06	−0.58	−0.13	0.07	0.16	0.57
UV Radiation	0.19	0.02	0.06	0.16	0.25	0.66
O_3_	0.29	0.06	0.18	0.25	0.38	1.43

## Data Availability

The data used in this study are available from the corresponding authors on request.

## Supplementary Materials

The following supporting information can be downloaded at: https://www.mdpi.com/article/10.3390/toxics14060537/s1, Figure S1: Distribution diagram of standardized residuals; Table S1: Parameters of XGBoost Model; Table S2: Parameters of Random Forest Model; Table S3: Fitting parameters of multiple linear regression model; Table S4: Residual Parameters of MLR Model; Table S5: Parameter results of GTWR model.
