# Supplementary material for: Interpretable Machine Learning and Spatiotemporal Modeling of Meteorological and Environmental Drivers for Tuberculosis Incidence in China
Source: Toxics. 2026 Jun 21;14(6):537. doi: 10.3390/toxics14060537 (PMC13307691; doi:10.3390/toxics14060537)
Supplement: Supplementary file 1 [file toxics-14-00537-s001.zip › toxics-4342731-supplementary.pdf]

Supplementary material for

Interpretable Machine Learning and Spatiotemporal Modeling of Meteorological and Environmental Drivers for Tuberculosis Incidence in China

Zihao Wang[1], Siyuan Li[*1], Xiaotong Jiang[*2], Kang Hu[3], Yangzhou Wu[4],

1 State Key Laboratory of Spatial Datum, College of Geographical Sciences, Henan University, Zhengzhou 450046, China; zihaowang@henu.edu.cn
2 Shandong Key Laboratory of Eco-Environmental Science for the Yellow River Delta, Shandong University of Aeronautics, Binzhou 256603, China
3 Jiangsu Collaborative Innovation Center of Atmospheric Environment and Equipment Technology, Nanjing University of Information Science & Technology, Nanjing 210044, China; 004066@nuist.edu.cn
4 School of Environment and Architecture, University of Shanghai for Science and Technology, Shanghai 200093, China; yangzhou@usst.edu.cn
* Correspondence: siyuanli@henu.edu.cn (S.L.); xtjiang@zju.edu.cn (X.J.)

Section S1

The tuberculosis (TB) incidence rate was set as the dependent variable, while meteorological and environmental factors—including surface pressure, high vegetation coverage, $PM_{2.5}$, relative humidity (RH), east west wind speed components (U), north south wind speed components (V), uv radiation, O_3, and temperature—were used as key independent variables. We constructed both the XGBoost regression model and the Random Forest model for comparative analysis.

The dataset was randomly split into an 80% training set and a 20% test set to ensure the rigor and fairness of the comparison. Hyperparameter configuration for each model was completed through a "global search followed by fine-tuning" process, guided by Bayesian optimization combined with 5-fold cross-validation to enhance the reliability of the optimized hyperparameters. The final optimized hyperparameter combinations (with specific settings for XGBoost detailed in Table S1 and for Random Forest in Table S2) were confirmed to achieve optimal model performance.

For the XGBoost model, the tuning process adopted Bayesian optimization and was conducted in sequence: first fixing the basic parameter (learning rate = 0.1), then optimizing decision tree structure parameters (max_depth, min_child_weight, gamma), adjusting feature/sample sampling parameters (colsample_bytree, subsample), and finally optimizing regularization parameters (reg_alpha, reg_lambda). The random seed (seed = 27) was fixed to ensure reproducibility, and the root mean square error (RMSE) was used as the evaluation metric. The final key hyperparameters that achieved optimal performance (shown in Table S1) are: n_estimators = 150, max_depth = 7, min_child_weight = 3, gamma = 0, colsample_bytree = 0.9, subsample = 0.6, reg_alpha = 0.22, reg_lambda = 900, eval_metric = RMSE, with the training set and test set proportions set to 0.8 and 0.2, respectively.

For the Random Forest model, to enhance the rationality of comparison with XGBoost, the tuning process adopted Bayesian optimization and referenced XGBoost's parameter direction: first aligning key parameters (n_estimators = 150, max_depth = 7, random_state = 27, and 80%/20% training/test set split), then independently optimizing adaptive parameters based on the ensemble characteristics of Random Forest. The final parameters that achieved optimal performance (shown in Table S2) are: min_samples_leaf = 3, min_samples_split = 2, max_features = 0.9, bootstrap = True, and criterion = mse.

The parameter configurations of both models ensure the rigor of the comparative experiment through consistent dataset splitting and key parameter alignment. They also adapt to the respective model logics via targeted tuning, achieving a balance between fitting performance and generalization ability while reaching optimal overall performance.

Table S1. Parameters of XGBoost Model.

Parameter	Value	Parameter	Value
Proportion of training set	0.8	Proportion of test set	0.2
learning_rate	0.1	colsample_bytree	0.9
n_estimators	150	subsample	0.6
max_depth	7	reg_alpha	0.22
min_child_weight	3	reg_lambda	900
gamma	0	seed	27
eval_metric	RMSE		

Table S2. Parameters of Random Forest Model.

Parameter	Value	Parameter	Value
Proportion of training set	0.8	Proportion of test set	0.2
n_estimators	150	max_features	0.9
max_depth	7	random_state	27
min_samples_leaf	3	min_samples_split	2
bootstrap	True	criterion	mse

Table S3. Fitting parameters of multiple linear regression model.

Model	R	R^2	R^2 Adjusted	Error in standard estimation	Debbin Watson value
MLR	0.440a	0.194	0.193	2.90119	1.598

Table S4 Residual Parameters of MLR Model.

	Min	Max	Mean	RSD	Number of cases
Estimate	1.7593	10.5147	5.9894	1.42063	5952
Residual	-8.02582	27.14930	0	2.89924	5952
Standard Predicted value	-2.978	3.185	0	1.000	5952
Standardized Residual	-2.766	9.358	0	0.999	5952

Table S5. Parameter results of GTWR model.

**Variable**	Value
Bandwidth	0.114996
Residual Squares	1423.63
Sigma	0.489066
AICc	8720.16
R^2	0.760815
R^2 Adjusted	0.760493
Spatio-temporal Distance Ratio	0.541833
Trace_of_SMatrix	165.457

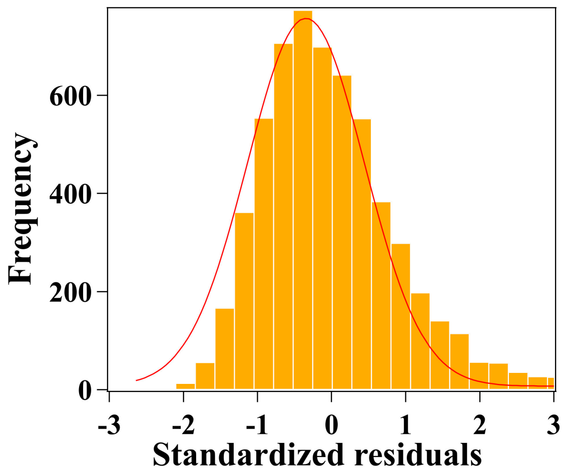

Figure S1. Distribution diagram of standardized residuals.
